# Attitudes towards vaccination of persons with mental disorders

**DOI:** 10.1192/j.eurpsy.2023.1251

**Published:** 2023-07-19

**Authors:** S. Djordjevic, L. Maodus, S. Kijac

**Affiliations:** Affective disorders, Special Hospital for Psychiatric Disease Kovin, Kovin, Cara Lazara 253, Kovin, Serbia

## Abstract

**Introduction:**

The effects of the COVID 19 pandemic manifests in all segments of society, and they have special impact on the vulnerable population of persons with mental disorders. After two years of pandemic, the vaccines against COVID 19 are still the most important strategy against infection, disease, transmission and reinfection.

**Objectives:**

The aim of our research was to compare the attitudes of different subcategories of people with mental disorders towards vaccination and to discover potential factors that could participate in the decision-making process.

**Methods:**

The research was conducted in the outpatients population, from January to March 2022, in Special Hospital for Psychiatric Disease Kovin, Serbia. All patients included in the research signed a voluntary consent to participate in the research. The data was collected from a self-assessment questionnaire, consisted of 4 segments: sociodemographic characteristics, a set of questions related to the COVID pandemic, history of influenza vaccination and attitudes towards vaccination.

**Results:**

The questionnaire included 147 people, 50 of whom were the control group of health professionals employed in SBPB “Kovin”, while in the subgroup of 97 respondents there were 49 people with a diagnosis of one of the non-psychotic disorders and 48 people with a diagnosis of one of the psychotic disorders. In the total population examined, the percentage of vaccinated was 53.97. In the population of persons with mental disorders, this percentage was lower compared to the control group. The results indicated that there is no statistically significant difference in attitudes towards vaccination in relation to the level of education. People who have been vaccinated are also people who would accept vaccination if another pandemic were to occur. In the unvaccinated subgroup, the greatest concern is vaccine safety. This group of respondents did not significantly differ from the vaccinated when it comes to fear of COVID 19 infection and concern about possible illness of family members.

**Conclusions:**

The research showed that slightly more than half of the examined population of persons with mental disorders who use the services of the psychiatric dispensary SBPB “Kovin” were vaccinated.

There is no statistically significant difference in the vaccination status against COVID 19 infection in the subcategories of persons with non-psychotic and psychotic symptoms. In the unvaccinated category, concerns about vaccine safety are a significant cause of vaccine refusal. Such preliminary results indicate the need to raise awareness and provide contoured, timely information and education regarding the pandemic of persons with mental disorders. Promotion of mental health and finding appropriate strategies in the provision of services to persons with mental disorders is one of the key tasks of the psychiatric service.

**Disclosure of Interest:**

None Declared

